# Effect of encapsulated edible halophyte with different biopolymers on the inhibition of sodium absorption in mouse

**DOI:** 10.1002/fsn3.2163

**Published:** 2021-02-16

**Authors:** Eun Young Jung, Seung Yun Lee, Da Young Lee, On You Kim, Yeonhwa Park, Sun Jin Hur

**Affiliations:** ^1^ Department of Animal Science and Technology Chung‐Ang University Anseong‐si Korea; ^2^ Department of Food Science University of Massachusetts Amherst Amherst MA USA

**Keywords:** biopolymer encapsulation, halophyte, inhibiting sodium absorption, mouse model

## Abstract

The purpose of this study was to investigate the effects of edible halophyte *Salicornia herbacea* encapsulated with biopolymers on inhibition of sodium absorption in mouse. *Salicornia herbacea* encapsulated with four biopolymers (pectin, chitosan, cellulose and dextrin) were fed to mice for 48 hr, and inhibiting sodium absorption was measured. In primary in vitro condition, fresh *Salicornia herbacea* encapsulated with 1% cellulose had 40% binding rate. Juice residue *Salicornia herbacea* encapsulated with 1% chitosan had the highest sodium binding rate by 50%. In mouse model, fresh, juice, and juice residue of *Salicornia herbacea* encapsulated with 4% chitosan had the highest sodium absorption inhibitory rate by 19%. These results indicate that biopolymer‐encapsulated *Salicornia herbacea* could be combined with sodium under in vitro condition, and *Salicornia herbacea* encapsulated with biopolymers reduced sodium absorption in a mouse model. Chitosan and cellulose had the highest sodium absorption inhibitory effects compared with the other biopolymers.

## Graphical abstract

Edible *Salicornia herbacea* (SH) encapsulated with biopolymer can bind Na+. SH encapsulated with chitosan and cellulose reduced sodium absorption in mouse. SH encapsulation with biopolymers can be applied to sodium reduction technology.

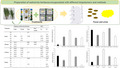

## 1 INTRODUCTION

Sodium is the principal cation in extracellular fluid in the body and is an essential nutrient necessary for maintenance of plasma volume, acid–base balance, transmission of nerve impulses and normal cell function (Organization, 2012). In healthy individuals, nearly 100% of ingested sodium is absorbed during digestion, and urinary excretion is the primary mechanisms for maintaining sodium balance. However, a high intake of sodium increases blood pressure and the risk of cardiovascular disease (Wang & Labarthe, 2011); thus, reduced dietary sodium intake has been recommended for at least a half century (Alderman & Cohen, 2012). Data from around the world suggest that the population average sodium consumption is well above the minimal physiological needs, and in many countries is above the value recommended by the 2002 Joint World Health Organization/Food and Agriculture Organization of the United Nations (WHO/FAO) Expert Consultation of 2 g sodium/day (equivalent to 5 g salt/day) (Brown et al., 2009; Organization, 2003). Therefore, reduction in sodium intake is beneficial to reduce the risk of cardiovascular disorders related to the high sodium intake. For that reasons, reduction in sodium intake has been extensively studied in food industries around the world. For instance, sodium replacer or saltiness enhancement such as SaltWise®, sub4salt®, Premier^TM^ Light Salt 50/50 Blend, SodiumSense^TM^ System V1 ~ V3, Salt Rite^TM^, AlgySalt®, Na‐K^TM^, Kasomel^TM^, Sodium gluconate, PELL^TM^ K Low Sodium Baking Powder, Reducit® 0402/20‐MG‐L, FlakeSelect^TM^ Flour Sea Salt, or monosodium glutamate has been developed around the world. In the mean times, Cermak et al. (2002) reported that ammonia inhibits sodium absorption in the proximal colon of rats and the effect is independent of the inhibition of the Na^+^‐H^+^ exchanger isoforms NHE^2^ (sodium‐proton‐exchanger subtype) and NHE^3^ and requires the presence of chloride ions (Cermak et al., 2002). Tenapanor is an inhibitor of the sodium proton (Na^+^‐H^+^) exchanger NHE3, which inhibited sodium uptake in the gastrointestinal tract (Spencer et al., 2014). Linz et al. (2016) reported that reduction in sodium absorption by NHE3 inhibition in the gut lowered high blood pressure (Linz et al., 2016). Although many sodium replacers and sodium enhancement have developed, no one can absolutely replace the natural functions of sodium (or salt) such as saltiness, flavor or antimicrobial activity. Therefore, we have developed a different approach to reduce sodium intake without reduction in natural sodium functions in foods, that is, inhibition of sodium absorption without sodium replacement or sodium reduction in foods after ingestion. Our several preliminary studies found that biopolymer encapsulation with halophyte decreased sodium absorption and increased sodium excretion without reduction in natural sodium function after their ingestion. Therefore, the aim of this study was to investigate the effects of edible halophyte *Salicornia herbacea* encapsulated with biopolymers on inhibition of sodium absorption in mouse, and to determine the binding rate of halophyte and various biopolymers under the in vitro condition.

## 2 MATERIALS AND METHODS

### 2.1 Preparation of four *Salicornia herbacea* solutions

Edible *Salicornia herbacea* was purchased from Suncheon salt farms (Suncheon‐si, Jeollanamdo, Korea), and four *Salicornia herbacea* samples were prepared as follows:

1. Fresh sample: fresh *Salicornia herbacea* was ground using blender for 1min.
2. Powder sample: Fresh *Salicornia herbacea* was dried at 70℃ for 48h and ground using blender for 1min.
3. Juice sample: Fresh *Salicornia herbacea* was squeezed using a juice extractor, and juice was collected.
4. Juice residue sample: Fresh *Salicornia herbacea* was squeezed, and residue of juice was collected.

**Figure 1:**
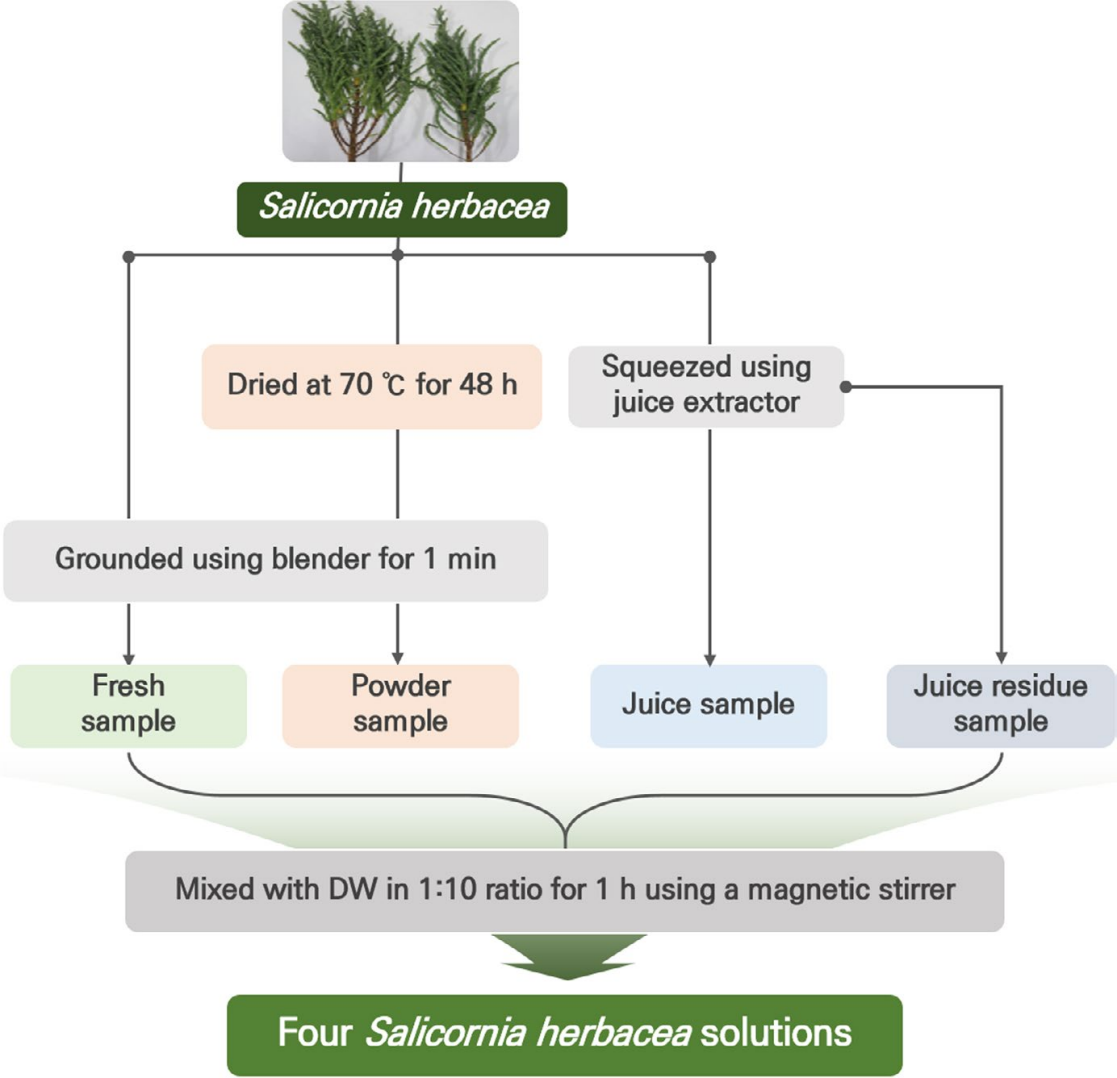
A schematic flow diagram of the four *Salicornia herbacea* solutions

Four *Salicornia herbacea* samples were mixed with DW in 1:10 ratio for 1hr using a magnetic stirrer (Figure 1).

### 2.2 Preparation of four biopolymer solutions

Four biopolymer (pectin, chitosan, cellulose, and dextrin) solution was prepared as follows:

1. For initial biopolymer solution preparation, four biopolymers were mixed with DW (deionized water) and final volume of four biopolymer solutions was 1 to 5% v/w, respectively.
2. Solutions were stirred for 12 hr using a magnetic stirrer to mixing.

### 2.3 Encapsulation of four *Salicornia herbacea* with four biopolymers

Four *Salicornia herbacea* (fresh, powdered, juice, and juice residue) solutions were mixed with four biopolymer solution using a magnetic stirrer for 30 min.

### 2.4 Sodium binding rate of *Salicornia herbacea* encapsulated with four biopolymers in an in vitro condition

The sodium binding rate of four *Salicornia herbacea* solutions with four biopolymer solutions was measured using digital salimeter (DX223, Metelo‐Toledo). Briefly, a probe of salimeter was placed into fifty mL of *Salicornia herbacea* encapsulated with biopolymer solution, and then, sodium concentration was analyzed.

$$\text{Sodium binding rate}\;\left(\%\right)=\frac{\text{Sodium concentration of before encapsulation}‐\text{Sodium concentration of after encapsulation}}{\text{Sodium concentration of before encapsulation}}\;\times 100.$$

### 2.5 Preparation of animal diet

**Figure 2:**
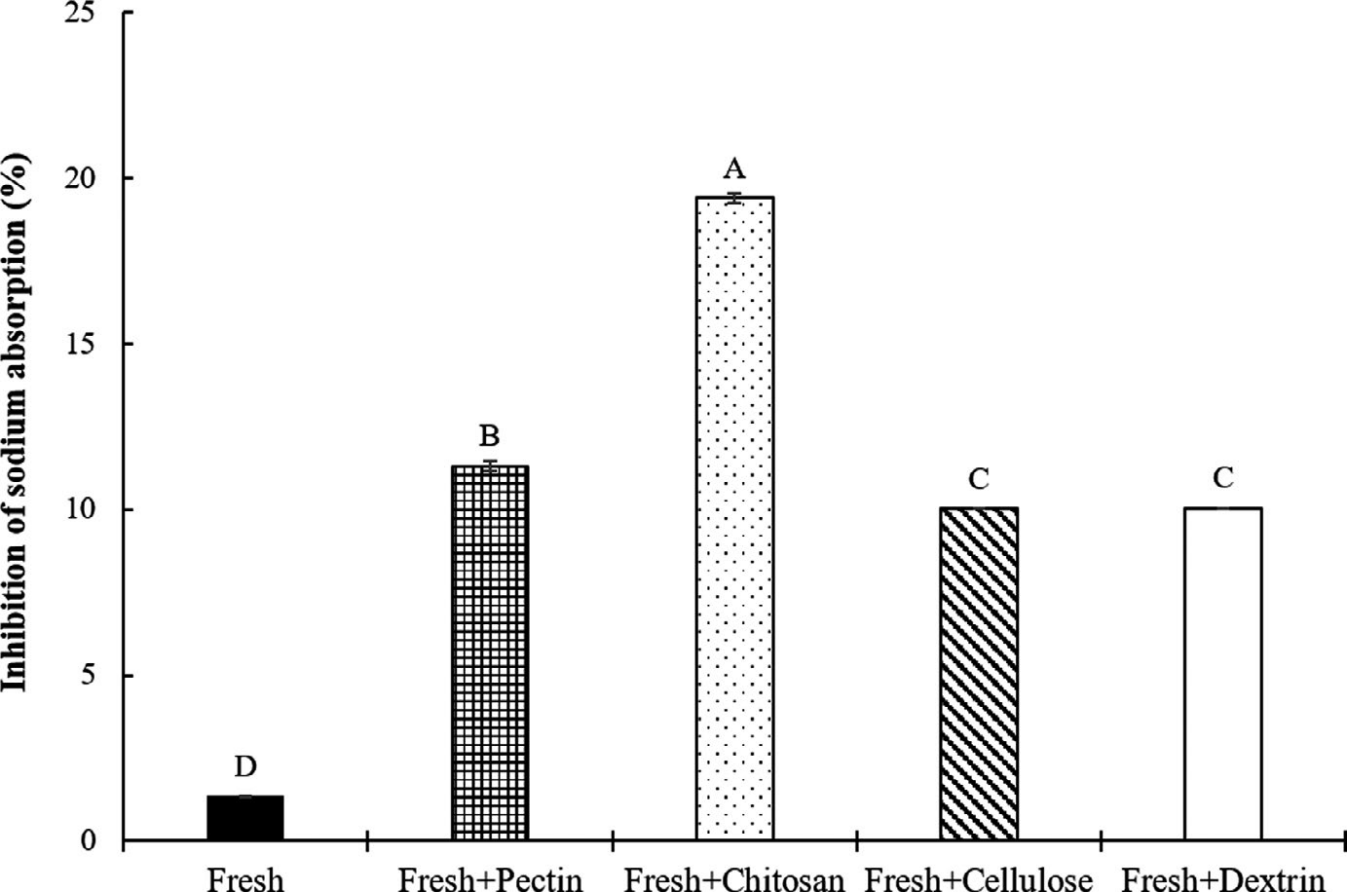
Effects of fresh *Salicornia herbacea* encapsulated with different biopolymers on the inhibition of sodium absorption in the mouse model. Fresh: commercial diet with 4% fresh; fresh + pectin: commercial diet with 4% fresh + 5% pectin; fresh + chitosan: commercial diet with 4% fresh + 5% chitosan; fresh + cellulose: commercial diet with 4% fresh + 5% cellulose; fresh + dextrin: commercial diet with 4% fresh + 5% dextrin. The data are presented as the mean values ± standard deviation. ^A‐D^ Means with different letters as superscripts indicate significant differences (*p* < .05)

The dietary groups were divided into 20 (4 experimental group × 5 treatments) and diet was prepared as follows:

First group (Figure 2) was divided into 5 treatments as follows:

‐ C: commercial diet with 4% fresh *Salicornia herbacea* without biopolymer
‐ Fresh + pectin: commercial diet with 4% fresh *Salicornia herbacea* encapsulated with 5% pectin
‐ Fresh + chitosan: commercial diet with 4% fresh *Salicornia herbacea* encapsulated with 5% chitosan
‐ Fresh + cellulose: commercial diet with 4% fresh *Salicornia herbacea* encapsulated with 5% cellulose
‐ Fresh + dextrin: commercial diet with 4% fresh *Salicornia herbacea* encapsulated with 5% dextrin

**Figure 3:**
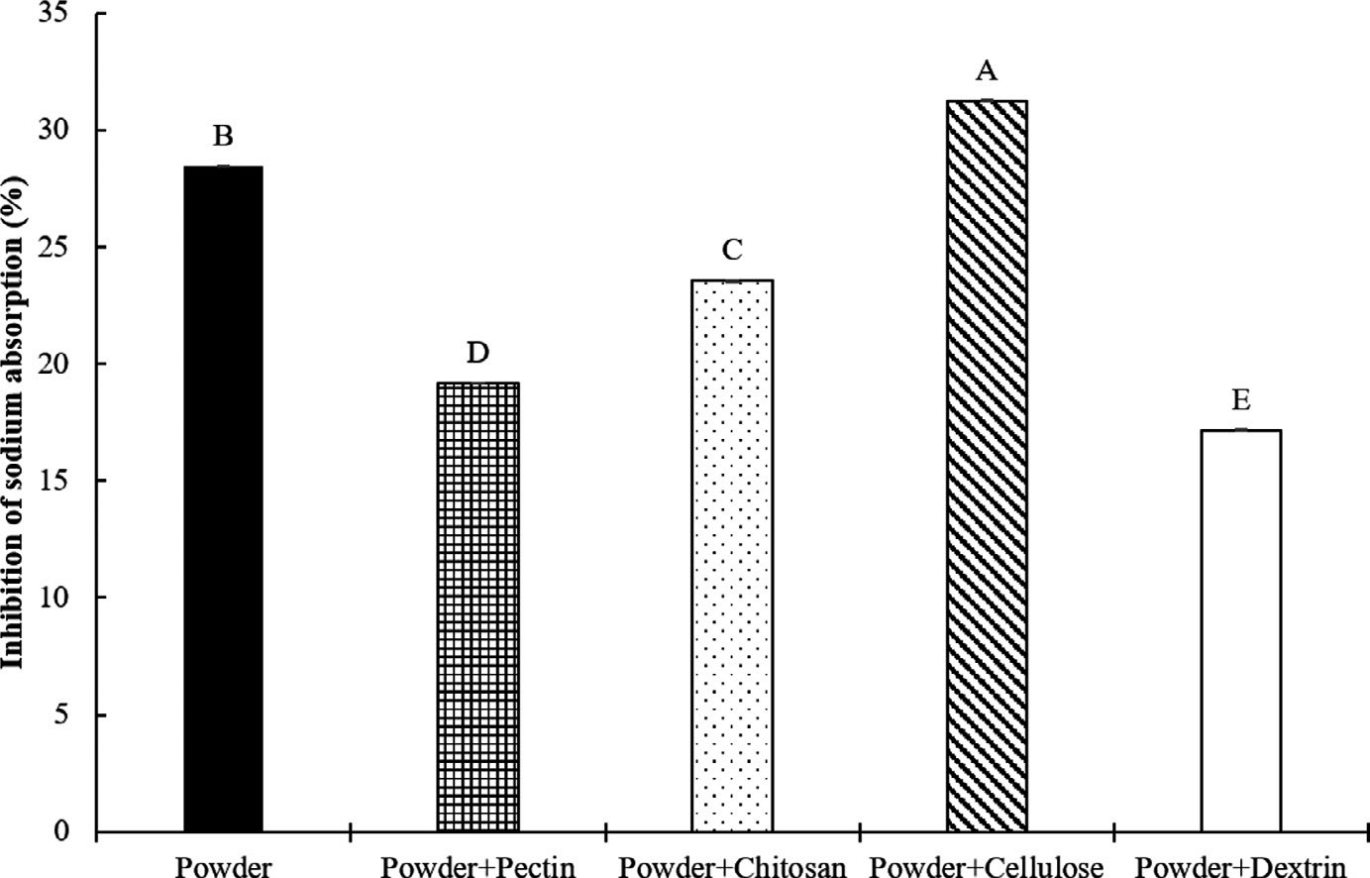
Effects of powder *Salicornia herbacea* encapsulated with different biopolymers on the inhibition of sodium absorption in mouse model. Powder: commercial diet with 4% powder; powder + pectin: commercial diet with 4% powder + 5% pectin; powder + chitosan: commercial diet with 4% powder + 5% chitosan; powder + cellulose: commercial diet with 4% powder + 5% cellulose; powder + dextrin: commercial diet with 4% powder + 5% dextrin. The data are presented as the mean values ± standard deviation. ^A‐E^ Means with different letters as superscripts indicate significant differences (*p* < .05)

Second group (Figure 3) was divided into 5 treatments as follows:

‐ Powder: commercial diet with 4% powder *Salicornia herbacea* without biopolymer
‐ Powder + pectin: commercial diet with 4% powder *Salicornia herbacea* encapsulated with 5% pectin
‐ Powder + chitosan: commercial diet with 4% powder *Salicornia herbacea* encapsulated with 5% chitosan
‐ Powder + cellulose: commercial diet with 4% powder *Salicornia herbacea* encapsulated with 5% cellulose
‐ Powder + dextrin: commercial diet with 4% powder *Salicornia herbacea* encapsulated with 5% dextrin

**Figure 4:**
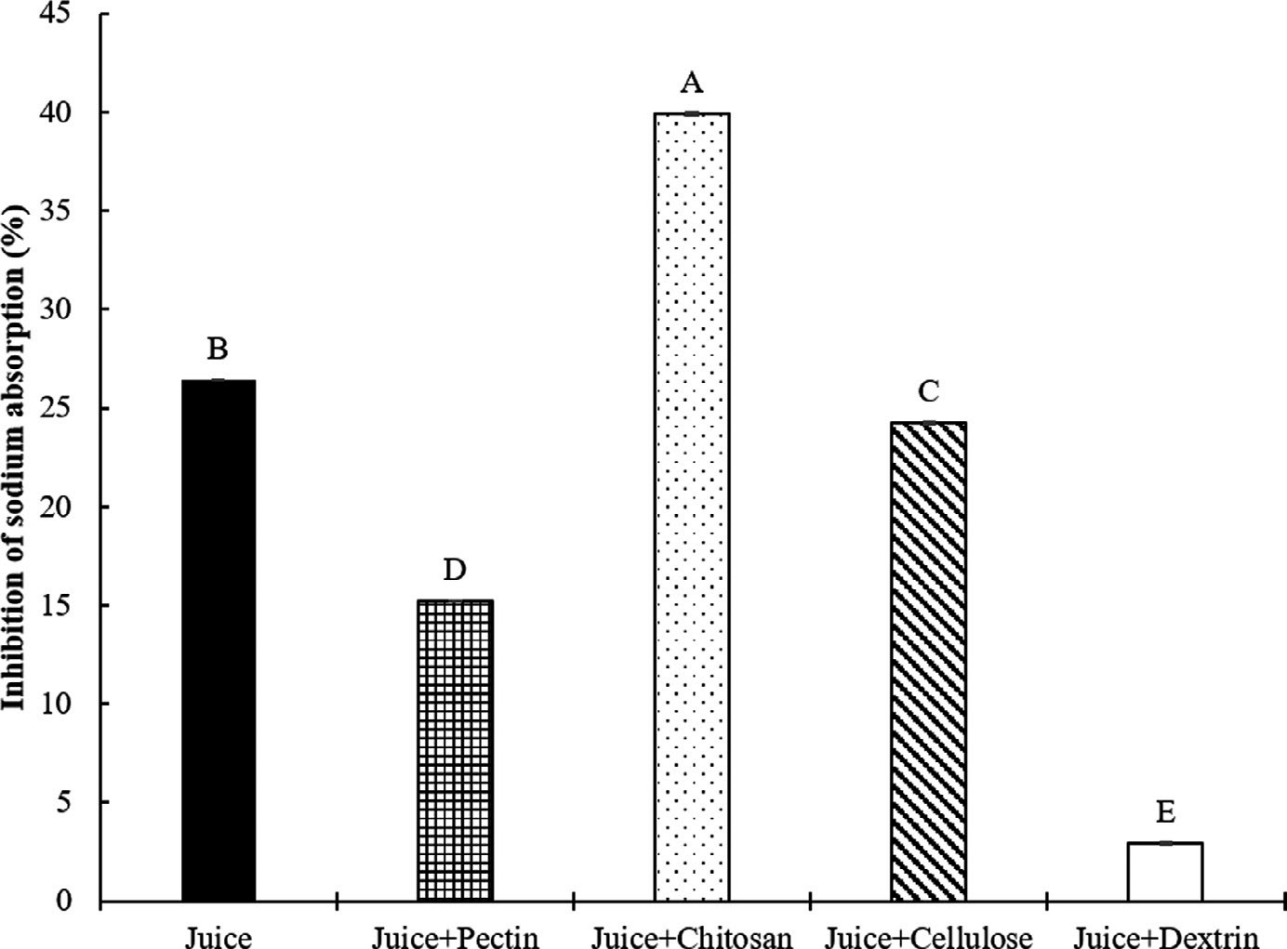
Effects of juice *Salicornia herbacea* encapsulated with different biopolymers on the inhibition of sodium absorption in the mouse model. Juice: commercial diet with 4% juice; juice + pectin: commercial diet with 4% juice + 5% pectin; juice + chitosan: commercial diet with 4% juice + 5% chitosan; juice + cellulose: commercial diet with 4% juice + 5% cellulose; juice + dextrin: commercial diet with 4% juice + 5% dextrin. The data are presented as the mean values ± standard deviation. ^A‐E^ Means with different letters as superscripts indicate significant differences (*p* < .05)

Third group (Figure 4) was divided into 5 treatments as follows:

‐ Juice: commercial diet with 4% juice *Salicornia herbacea* without biopolymer
‐ Juice + pectin: commercial diet with 4% juice *Salicornia herbacea* encapsulated with 5% pectin
‐ Juice + chitosan: commercial diet with 4% juice *Salicornia herbacea* encapsulated with 5% chitosan
‐ Juice + cellulose: commercial diet with 4% juice *Salicornia herbacea* encapsulated with 5% cellulose
‐ Juice + dextrin: commercial diet with 4% juice *Salicornia herbacea* encapsulated with 5% dextrin

**Figure 5:**
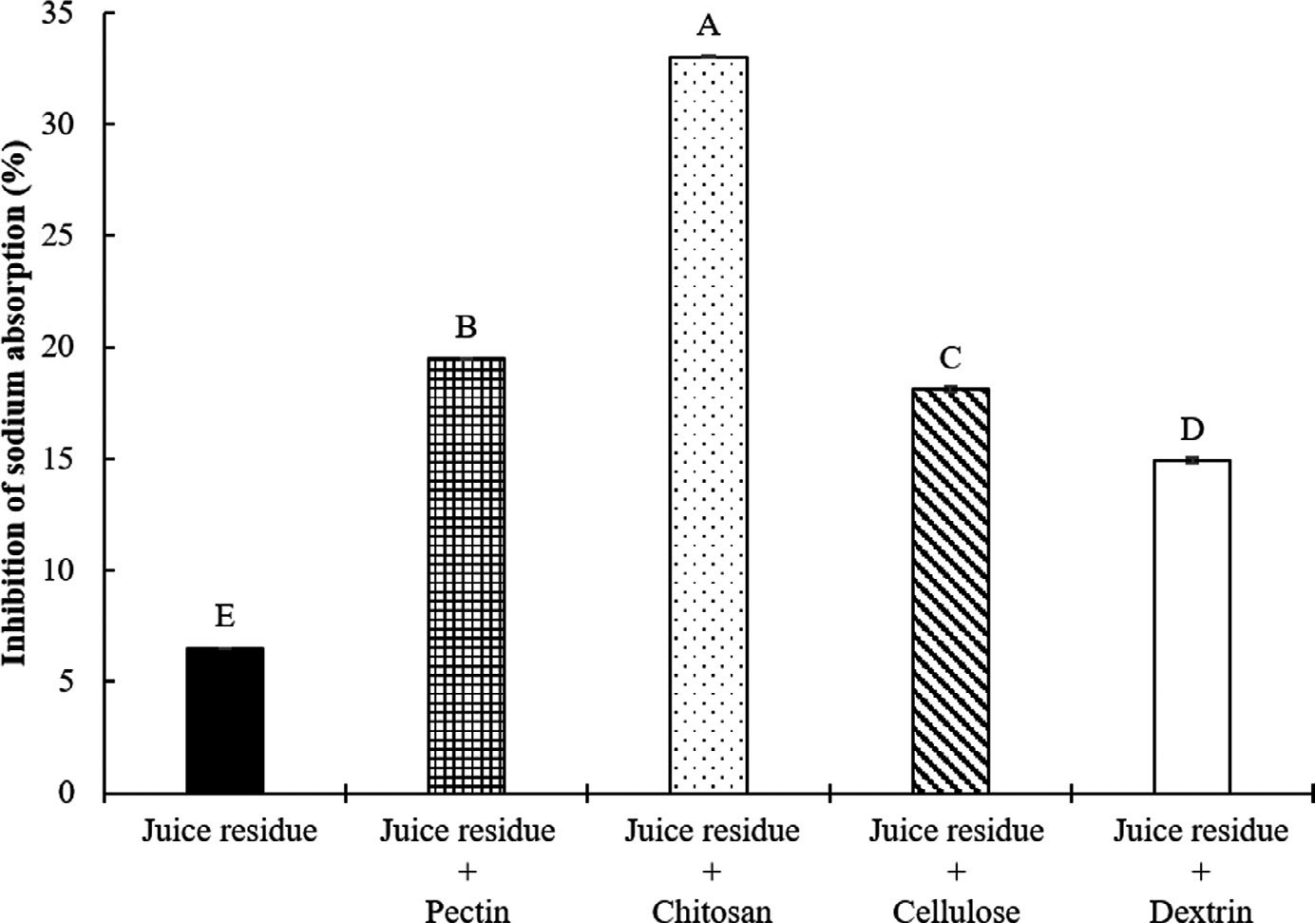
Effects of juice residue *Salicornia herbacea* encapsulated with different biopolymers on the inhibition of sodium absorption in the mouse model. Juice residue: commercial diet with 4% juice residue; juice residue + pectin: commercial diet with 4% juice residue + 5% pectin; juice residue + chitosan: commercial diet with 4% juice residue + 5% chitosan; juice residue + cellulose: commercial diet with 4% juice residue + 5% cellulose; juice residue + dextrin: commercial diet with 4% juice residue + 5% dextrin. The data are presented as the mean values ± standard deviation. ^A‐E^ Means with different letters as superscripts indicate significant differences (*p* < .05)

Fourth group (Figure 5) was divided into 5 treatments as follows:

‐ Juice residue: commercial diet with 4% juice residue *Salicornia herbacea* without biopolymer
‐ Juice residue + pectin: commercial diet with 4% juice residue *Salicornia herbacea* encapsulated with 5% pectin
‐ Juice residue + chitosan: commercial diet with 4% juice residue *Salicornia herbacea* encapsulated with 5% chitosan
‐ Juice residue + cellulose: commercial diet with 4% juice residue *Salicornia herbacea* encapsulated with 5% cellulose
‐ Juice residue + dextrin: commercial diet with 4% juice residue *Salicornia herbacea* encapsulated with 5% dextrin

### 2.6 Animal feeding experiment

One hundred female ICR mice were purchased from Orient Bio (Seongnam‐si, Gyeonggi, Korea). The mice were maintained under rested conditions for 1 weeks before experimental feeding. The mice were housed in individual cages in a windowless room with a 12/12‐hr light/dark cycle, in accordance with the rules of the Institutional Animal Care and Use Committee of Chung‐Ang University (IACUC approval number: 202000050). Water was provided ad libitum during the experiment. After 1 week of adaptation, one hundred mice were randomly divided into 20 groups (*n* = 5 mice per group). For analysis of inhibition of sodium absorption in the mouse model, the experimental diets were fed for 48hr in mouse models. After 48hr of experimental diet feeding, all feces and urine of mice were collected, and then, sodium concentration was measured. This feeding experiment was replicated three times (100 mice × 3 times experiment) with 1‐week interval.

### 2.7 Analysis of inhibition of sodium absorption in the mouse model

Inhibition of sodium absorption rate (%) in mouse models was determined with different sodium concentrations between consumed sodium in diet and sodium concentration in feces and urine of mice. To determine inhibition of sodium absorption rate, animal diet was mixed with DW in 1:2 ratio and feces and urine of mice were mixed with DW in 1:2 ratio, and then, sodium concentration was measured using digital salimeter (DX223, Metelo‐Toledo, Switzerland), respectively. The measurement was replicated five times.

$$\text{Inhibition of sodium absorption}\;\left(\%\right)=\frac{\text{Sodium concentration of consumed animal diet}\;‐\text{Sodium concentration of animal feces and urine}}{\text{Sodium concentraion of consumed animal diet}}\times 100.$$

### 2.8 Statistical analysis

Statistical analyses were performed for three independent batches of samples, and a total of one hundred mice were used for analysis with three time repetitions. The data for each batch regarding inhibition of sodium absorption were analyzed using ANOVA with the SAS software (SAS Inst. Inc.). Significant differences (*p* < .05) between the mean values for different groups were determined for inhibition of sodium absorption (*n* = 5).

## 3 RESULTS

The effects of *Salicornia herbacea* encapsulated with biopolymers on the sodium binding rate under in vitro condition were determined and are present in Table 1. To determine sodium binding rate under in vitro condition, four *Salicornia herbacea* samples (fresh, powder, juice, and juice residue) and two sodium chloride samples (1% and 5%) were encapsulated with four biopolymers, for example, pectin, chitosan, cellulose, and dextrin, respectively. Fresh *Salicornia herbacea* encapsulated with 1% cellulose had higher sodium binding rate by 40%. Powder *Salicornia herbacea* encapsulated with 4% cellulose shown to have 28.57% sodium binding rate. Sodium binding rate of juice *Salicornia herbacea* encapsulated with 3% pectin was 12.89%. Juice residue *Salicornia herbacea* encapsulated with 1% chitosan had highest sodium binding rate by 50%. 1% NaCl encapsulated with 5% pectin showed 37.50% sodium binding rate. 5% NaCl encapsulated with 5% dextrin showed 44.44% sodium binding rate. To compare the sodium binding rate among the biopolymers, cellulose had great binding ability with various *Salicornia herbacea* samples and regardless of NaCl concentration, except juice and juice residue of *Salicornia herbacea*. However, dextrin had less binding effect compared with other biopolymer in most *Salicornia herbacea* samples.

**Table 1:** Effects of *Salicornia herbacea* encapsulated with different biopolymers on the sodium binding rate in an in vitro condition

Treatments		Solutions of *Salicornia herbacea* ^a^				NaCl	
		Fresh^b^	Powder^b^	Juice^b^	Juice residue^b^	1%	5%
Pectin^c^	1%	‐	‐	3.17	‐	‐	‐
	2%	‐	‐	‐	‐	‐	3.13
	3%	20.00	0.37	12.89	‐	28.57	36.84
	4%	‐	7.45	‐	‐	14.29	18.18
	5%	‐	6.62	‐	‐	37.50	21.88
Chitosan^c^	1%	33.33	‐	8.88	50.00	‐	12.05
	2%	33.33	‐	4.53	50.00	‐	6.11
	3%	33.33	‐	‐	‐	13.10	6.43
Cellulose^c^	1%	40.00	15.38	‐	‐	28.57	10.00
	2%	20.00	14.29	‐	‐	16.67	23.33
	3%	33.33	15.38	8.70	‐	14.29	15.63
	4%	20.00	28.57	‐	‐	28.57	6.67
	5%	20.00	7.14	‐	‐	14.29	10.00
Dextrin^c^	1%	‐	‐	10.63	‐	‐	16.67
	2%	‐	‐	‐	‐	‐	5.71
	3%	‐	‐	‐	‐	‐	‐
	4%	‐	‐	3.70	‐	‐	6.67
	5%	‐	‐	‐	‐	‐	44.44

^a^ To make *Salicornia herbacea* encapsulated with biopolymers, four *Salicornia herbacea* solutions (1:10 ratio with DW) and two NaCl were mixed with different biopolymer (pectin, chitosan, cellulose, and dextrin) solutions for 30min.

^b^ *Salicornia herbacea* samples were prepared as follows: fresh was ground using blender; powdered was dried at 70℃ for 48h and ground; juice was squeezed using a juice extractor, and residue of juice was collected.

^c^ Biopolymers (pectin, chitosan, cellulose, and dextrin) made solution to a final volume of 1–5% v/w.

The effects of the dietary *Salicornia herbacea* encapsulated with four biopolymers on the inhibition of sodium absorption in mice are shown in Figures 2, 3, 4, 5. To determine inhibition of sodium absorption rate in mice, each *Salicornia herbacea* (fresh herb, powder, juice, and juice residue) sample for control (commercial diet with 4% *Salicornia herbacea* samples) and four *Salicornia herbacea* samples encapsulated with four biopolymers (pectin, chitosan, cellulose, and dextrin) (commercial diet with 4% *Salicornia herbacea* samples + 5% biopolymers) were fed for 48hr in mouse models, and then, the concentration of sodium in consumed diets and feces and urine of mice was analyzed. The inhibition of sodium absorption of fresh *Salicornia herbacea* encapsulated with chitosan had around 14 times higher (19.41%) compared with non‐biopolymer‐encapsulated fresh *Salicornia herbacea* (1.36%) (Figure 2). Juice and juice residue *Salicornia herbacea* encapsulated with chitosan had also high inhibition of sodium absorption (1.5 and 5 times, respectively) compared with non‐biopolymer‐encapsulated juice and residue *Salicornia herbacea* (Figures 4 and 5). In powdered *Salicornia herbacea* encapsulated with four different biopolymer samples, inhibition of sodium absorption was 1.1 times higher in cellulose‐encapsulated sample than nonbiopolymer samples (Figure 3). However, inhibition of sodium absorption of dextrin was lower than that of other biopolymers among the biopolymer‐encapsulated *Salicornia herbacea* samples. As a result of this study, the authors can summarize that the effects of biopolymer encapsulation on the sodium binding rate of *Salicornia herbacea* samples under in vitro condition, cellulose had great combining ability with various *Salicornia herbacea* samples. Juice residue *Salicornia herbacea* had the best sodium binding rate with 1% chitosan. Also, the results of inhibition of sodium absorption in mouse model, fresh, juice, and residue *Salicornia herbacea* encapsulated with chitosan had great inhibition of sodium absorption, it ranged from 1.5 to 14 times. These results indicate that chitosan and cellulose were found to be more effective for the sodium binding and the inhibition of sodium absorption than other biopolymers in both of in vitro and mouse model.

## 4 DISCUSSION

Biopolymers such as chitosan, pectin, cellulose, or dextrin are known to bind inorganic ion strongly, and these are abundant nontoxic and edible polymers in nature and are appeared to be more economically attractive sorbent for inhibition of sodium absorption. Although the exact mechanisms for inhibition of sodium absorption of *Salicornia herbacea* encapsulation with biopolymers are still unclear, the authors would suggest several possible mechanisms. The first possible mechanism would be gelation properties of biopolymers. In this study, biopolymers such as pectin and chitosan bound with Na^+^ during encapsulation in terms of gelation properties. Chitosan and pectin can gel in the presence of divalent cations such as sodium and calcium, and this gelation is due to the formation of intermolecular junction zones between different chains. Muzzarelli (1983) also reported that chitosan is effective in the uptake of transition metal since the amino groups on chitosan chains serve as coordination sites (Muzzarelli, 1983). The adhesive properties of chitosan in a swollen state have shown to persist well during repeated contacts of chitosan and the substrate, which implied that, in addition to the adhesion by hydration, many other mechanisms, such as hydrogen bonding and ionic interactions, might also have been involved (Lehr et al., 1992). In general, three steps are involved in the encapsulation of bioactive agents: 1) the formation on the wall around the materials to be encapsulated; 2) ensuring that undesired leakage does not occur; and 3) ensuring that undesired materials are kept out (Gibbs, 1999; Mozafari et al., 2008). Biopolymers are capable of trapping Na^+^ in the gastrointestinal tract, and gel matrix formed by biopolymer is excreted in the feces. This is why biopolymer encapsulation reduced sodium absorption in mouse models. Second possible mechanism would be resulted in increased solution viscosity. In general, an increase in solution viscosity is responsible for a decreased transit time and digestion rate of the ingested food in the gastrointestinal tract (Hur et al., 2013). Our previous study found that dietary biopolymers increased the viscosity and decreased digestion of the contents of the small intestine (Hur et al., 2013). In this study, biopolymer‐encapsulated *Salicornia herbacea* increased viscosity and decreased diffusion of sodium ion in the gastrointestinal tract of mice and subsequently decreased sodium absorption in mice. Third possible mechanism would be electrostatic interaction of biopolymers. Chitosan has been insisted that chitosan entraps lipids in the intestine, because of its cationic nature (Hur et al., 2009; Kanauchi et al., 1995). Several studies reported that the positive charge on chitosan has been found for its enhanced bioadhesion to negatively charged cell membranes. This enables the site‐specific applications in controlled delivery systems (Aksungur et al., 2004; He et al., 1998; Şenel et al., 2000). The authors assume that the positive charged chitosan under the acidic condition and negative charged chitosan under the alkali condition could be combined with Na^+^ although ionic crosslink of chitosan is depending on pH and degree of acetylation. Chitosan has been shown to form complexes with a large number of different polyanions, such as DNA, alginates, pectins, xanthan, glucosaminoglycans, and carboxymethyl cellulose (Nilsen‐Nygaard et al., 2015). Several studies also found that cellulose is attractive sorbent in terms of Na^+^ ion binding. Deshpande et al. (2008) reported that the interaction between the positively charged Na ion and the partially negatively charged OH groups on cellulose is found to be purely ionic (Deshpande et al., 2008). Na^+^ ions can combine with cellulose because hydrated alkali ions (Na^+^) present in aqueous systems swell the cellulose by penetration into it and also an exchange of hydrated shell with OH groups of cellulose can occur. Electrostatic interaction between Na^+^ ions and cellulose can occur in the absence of ligand in the solution. Therefore, edible dietary fibers in *Salicornia herbacea* and Na^+^ ion could be combined with electrical charged chitosan and cellulose in this study. As result of this study, the authors assume that possible mechanism for inhibition of sodium absorption by *Salicornia herbacea* encapsulated with biopolymers is believed to originated from electrostatic attractive of chitosan or cellulose in the gastrointestinal tract of mice.

## 5 CONCLUSIONS

This study determined the effect of edible *Salicornia herbacea* encapsulation with biopolymers on the sodium binding under in vitro condition and inhibition of sodium absorption in mouse models. As a result of this study, the authors found that biopolymer‐encapsulated *Salicornia herbacea* could be combined with sodium under in vitro condition, and *Salicornia herbacea* encapsulated with biopolymers reduced sodium absorption in the mouse model. In particular, chitosan and cellulose had greater effect of inhibition of sodium absorption both on in vitro condition and mouse model. Although data are not shown, our preliminary study found that inhibition of sodium absorption effect of biopolymers was higher in *Salicornia herbacea*‐encapsulated samples than pure sodium chloride encapsulated with biopolymer samples. Therefore, this approach, that is, *Salicornia herbacea* encapsulation with biopolymer, can be applied to one of the sodium reduction technologies. However, sodium not only plays an important role for saltiness and flavor in food stuff but also affects the control of microbial growths in foods during storage. Therefore, further studies are required to understand how to improve the effect of inhibition of sodium absorption without negative effect of sodium reduction in terms of food flavor and microbial growth.

## CONFLICT OF INTEREST

The authors have no conflict of interest to declare.

## ETHICAL APPROVAL

This study was approved by the Institutional Animal Care and Use Committee of Chung‐Ang University (IACUC approval number: 202000050).
